# Aroma enhancement and enzymolysis regulation of grape wine using *β*-glycosidase

**DOI:** 10.1002/fsn3.84

**Published:** 2014-01-13

**Authors:** Feng-Mei Zhu, Bin Du, Jun Li

**Affiliations:** 1 College of Food Science and Technology, Hebei Normal University of Science and TechnologyQinhuangdao, 066600, China; 2 Analysis and Testing Center, Hebei Normal University of Science and TechnologyQinhuangdao, 066600, China

**Keywords:** Aroma enhancement, enzymolysis regulation, gas chromatography-mass spectrometry, grape wine, Kramer sensory evaluation

## Abstract

Adding *β*-glycosidase into grape wine for enhancing aroma was investigated using gas chromatography-mass spectrometry (GC-MS) and Kramer sensory evaluation. Compared with the extract from control wines, the extract from enzyme-treated wines increased more aromatic compounds using steam distillation extraction (SDE) and GC-MS analyses. Theses aromatic compounds were as follows: 3-methyl-1-butanol formate, 3-pentanol, furfural, 3-methyl-butanoic acid, 2-methyl-butanoic acid, 3-hydroxy-butanoic acid ethyl ester, hexanoic acid, hexanoic acid ethyl ester, benzyl alcohol, octanoic acid, octanoic acid ethyl ester, dodecanoic acid, and ethyl ester. The enzymolysis regulation conditions, including enzymolysis temperature, enzymolysis time, and enzyme amount, were optimized through L_9_(3^4^) orthogonal test. Kramer sensory evaluation was performed by an 11-man panel of judges. The optimum enzymolysis regulation conditions were found to be temperature of 45°C, enzymolysis time of 90 min, and enzyme amount of 58.32 U/mL grape wine, respectively. The Kramer sensory evaluation supported that the enzyme-treated wines produced a stronger fragrance.

## Introduction

Grape is a non-climacteric fruit of the genus *Vitis* that grows on the perennial and deciduous woody vines. There are about 60 species of *Vitis*, which are mainly found in the temperate zones of the Northern Hemisphere and almost equally distributed between America and Asia (Mullins et al. 1992). Grape wine is an alcoholic beverage, typically made of fermented grape juice. Its composition and properties are related to the wine's origin and age. The constituents of wine are water, ethanol, saccharides, amino acids, phenolic compounds, and other pigments, and trace metal (Monaci et al. 2003; Roig and Thomas 2003; Katalinic et al. 2004; Nilsson et al. 2004).

Grape wine aromas are made up of several hundreds of volatile compounds. The aroma potential of grape wine derives from aromatic free volatiles and from non-volatile, odorless precursor, which may be hydrolyzed during the winemaking process (Villena et al. 2006). Grape-derived aroma and flavor compounds are present as free volatiles and, in part, as sugar-bound precursor including glycosides (McMahon et al. 1999). Glycosides which contain aroma and flavor aglycones may affect wine quality after hydrolysis. The liberation of free aglycones from glycosides is performed by acid-catalyzed reactions or by the action of endogenous *β*-glycosidase (EC.3.2.1.21; Bothlho et al. 2007). The release of volatile compounds from their glycoconjugates by acid catalysis during wine conservation is quite slow. The rapid release of aglycones from glycoconjugates, through the use of exogenous glycosidase, can thereby accelerate the formation of odor-active volatiles during conservation due to the reactivity of liberated aglycones in wine pH. Winemaking is a biotechnological process in which the use of exogenous enzyme preparations helps to solve the problem of the insufficient activity of endogenous activity in the grapes. The potential aromatic compounds in grape wine can be liberated by hydrolysis of *β*-glycosidase. Therefore, the aroma of grape wine is enhanced and the quality of grape wine is improved. It would be very available to study the aroma enhancement using *β*-glycosidase for grape wine. Due to the limited effect of glycosidase from grape and *Saccharomyces cerevisiae* in winemaking, a large part of glycosides is still present in young wines (Cabaroglu et al. 2003). Therefore, increasing interest has been devoted in the past years to the study of using exogenous glycosidase from yeast and from filamentous fungi to enhance wine aroma.

Some biotechniques have been of fundamental importance in oenology, among these technological innovations, enzymatic treatments by commercial preparation in free or immobilized form, selected yeasts, improvement of microbial starters, and enzyme immobilization (Palmer and Spagna 2007). But the use of the enzymes in the wine industry remains limited for several reasons that can be summarized as follows: traditionalism of winemakers, influence on enzymatic activities related to physicochemical characteristics of musts and wines (pH, temperature, ethanol, sugars, polyphenols, etc.) on enzymatic activities (Colagrande et al. 1994). The aromatic component of a wine is, moreover, closely related to its sensory quality, which is determined by the consumer's acceptability (Varela and Gambara 2006; Vilanova 2006). Recently, sensory analysis has defined its role in the oenological industry identifying the causes of variation in perceived quality, the corrective actions, thereby becoming instrument of quality control (Verzera et al. 2008). The main aim of this work was to investigate the release of glycosidically bound aroma compounds by adding exogenous *β*-glycosidase into wines and using gas chromatography-mass spectrometry (GC-MS) analyses and Kramer sensory evaluation.

## Materials and Methods

### Sampling

Two samples of Cabernet Sauvignon dry red wine, one was obtained from the Experimental Winery at the College of Food Science and Technology, Hebei Normal University of Science and Technology, the other was obtained from the Langgesi (Qinhuangdao) Ltd. Company (Changli County, Hebei Province, China).

The crude *β*-glycosidase obtained from our laboratory (Zhu et al. 2010) was added into two wines. The wine without adding enzymes was used as control.

### Microorganism

*Aspergillus oryzae* 3.481 and *Aspergillus niger* 3.316 strain were obtained from China General Microbiological Culture Collection Center. The fused protoplasts of *A. oryzae* 3.481 and *A. niger* 3.316 have been regenerated on regeneration medium containing (in g L^−1^): sucrose 0.5, glucose 2.0, peptone 2.0, yeast extract 1.0, and agar 20.0. The fusion strain was selected for further studies.

### Inoculum

The protoplasts of *A. oryzae* and *A. niger* high-producing *β*-glycosidase were prepared, formed, regenerated, and fused for screening strain, which were attained by our laboratory (Zhu et al. 2010).

### Enzyme assay

*β*-Glycosidase activities were determined in duplicate, using 0.5% (w/v) salicin as substrate. Assays were performed in acetate buffer pH4.8 and incubated at 65°C for 20 min. The amount of released glucose was determined by 3,5-dinitrosalicylic acid (DNS) reagent method. *β*-Glycosidase activity is expressed in units (U/mL), defined as micromoles of salicin hydrolyzed by *β*-glycosidase per minute, under the conditions of assay.

### Enzyme treatment

The wine samples added with *β*-glycosidase were kept in water bath oscillator for heating oscillation. The L_9_(3^4^) orthogonal design was arranged (Tables 1, 2) with the three independent variables of enzymolysis temperature, enzymolysis time, and enzyme amount.

**Table 1:** Orthogonal test.

Level	Temperature (°C)	Time (min)	Enzyme volume (mL)
1	40	30	8
2	45	60	10
3	50	90	12

**Table 2:** Sample number of L_9_(3^4^) orthogonal test.

Number	Temperature (°C)	Time (min)	Enzyme volume (mL)	Sample code
1	1 (40)	1 (30)	1 (8)	B
2	1 (40)	2 (60)	2 (10)	C
3	1 (40)	3 (90)	3 (12)	D
4	2 (45)	1 (30)	2 (10)	E
5	2 (45)	2 (60)	3 (12)	F
6	2 (45)	3 (90)	1 (8)	G
7	3 (50)	1 (30)	3 (12)	P
8	3 (50)	2 (60)	1 (8)	O
9	3 (50)	3 (90)	2 (10)	N

### Aromatic components extraction

The minor volatile components in grape wine were fractionated using the simultaneous distillation extraction (SDE) technique (Cai et al. 2001). Sample concentration was carried out by using a SDE apparatus. A quantity of 100 mL of wine was heated in a 500 mL round-bottomed flask. The liquid phase extractions were achieved and continuous reflux of water was maintained during the extraction time (1.5 h). The distillate was extracted three times with 200 mL anhydrous ether and combined. Extracts were desiccated over anhydrous sodium sulfate, filtered, and concentrated using a rotary evaporator. Finally, the concentrates were kept in vials at 4°C until GC-MS analysis.

### Aromatic components analysis: GC-MS

The GC-MS system comprised a gas chromatograph (model HP6890) and a Saturn mass spectrometer (Hewlett Packard, Palo Alto, CA). Separation was performed through a cross-linked polymethylsiloxane capillary column (HP-5, 30 m × 0.25 mm × 0.25 μm). Carrier gas was helium with a flow rate of 1 mL/min. The injector (the spilt flow ration was 40:1) was set to 230°C. The GC temperature program was 60°C, 5°C/min to 200°C, held for 10 min. The mass spectrometer was operated in the electron impact (EI) mode at ionization energy of 70 eV and the mass range scanned was 20–500 amu in the full scan acquisition mode. The temperature of EI mode was 230°C. The ion trap was set to 150°C. Mass spectrometer with an electron bombardment source was used for the sample analysis.

Major volatile compounds were analyzed by direct injection of 0.5 μL. Volatile compounds were identified by the NIST and WILEY Mass Spectral Search Program. The identification of the volatile compounds was confirmed by comparing the retention indices with standard values of authentic samples. The relative content was calculated from the area ratio. Following equations were used to determine their percentage concentration of the identified compounds (w/w):


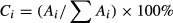


where *C* is concentration of one compound, *A* is peak area counts, Σ*A* is summation of all peak area counts, and subscript *i* represents one component.

### Sensory analysis

Wines were assessed by 11 judges from College of Food Science and Technology, Hebei Normal University of Science and Technology, Qinhuangdao, China, who had previous experience in wine sensory analysis. Assessment took place in a standard sensory-analysis chamber (ISO 8589, 2007) equipped with separate booths. All evaluations were conducted from 10.00 to 12.00 am in individual booths illuminated with white light. Water was provided for rinsing between wines. The order of presentation was randomized among judges and sessions. Triangle tests were performed to determine if the control samples and nine enzyme-treated wines from L_9_ (3^4^) were significantly different and the significance of the test was established from the statistical tables.

### Statistical analysis

The statistical significance of the effect of enzyme treatment on free and bound volatiles analyzed in triplicate was determined by analysis of variance (ANOVA) using the following software packages: SPSS 11.5 (SPSS Inc., Chicago, IL) for Windows statistical package.

## Results and Discussion

### GC-MS results

**Figure 1:**
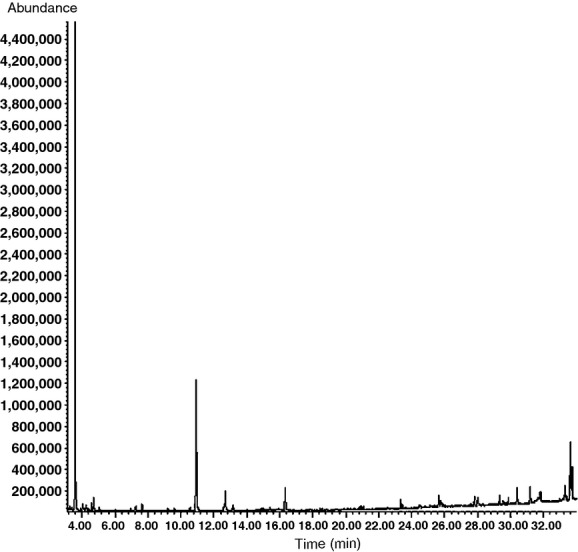
Total ionic chromatography of free aromatic components from control wines.

**Figure 2:**
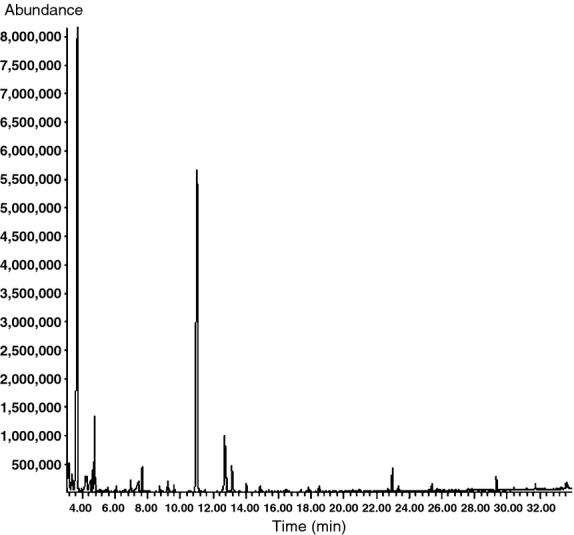
Total ionic chromatography of aromatic components from bound compounds with enzymolysis.

The aromatic components from control wine were analyzed by GC-MS, the total ionic chromatography is shown in Figure 1 and the relative content is shown in Table 3. The volatiles of wines were dominated by esters and alcohols, representing almost 90% of the volatiles (Table 3). The aromatic components from bound compounds with enzymolysis were analyzed by GC-MS, the total ionic chromatography is shown in Figure 2 and the relative content is shown in Table 4.

**Table 3:** GC-MS identification and relative content of free aromatic components from control wine.

Number	Retention time (min)	Chemical constituent	Formula	Molecular weight	Relative content (%)
1	3.55	Propanoic acid, 2-hydroxy-, ethyl ester	C_5_H_10_O_3_	118.13	64.80
2	4.57	1-Hexanol	C_6_H_14_O	102.18	0.70
3	4.71	1-Butanol, 3-methyl-, acetate	C_7_H_14_O_2_	130.18	2.23
4	4.76	1-Butanol, 2-methyl-, acetate	C_7_H_14_O_2_	130.18	0.23
5	5.53	Butyrolactone	C_4_H_6_O_2_	86.09	0.15
6	11.25	Phenylethyl alcohol	C_8_H_10_O	122.16	17.80
7	12.78	Butanedioic acid, diethyl ester	C_8_H_14_O_4_	174.20	3.29
8	14.92	Acetic acid, 2-phenylethyl ester	C_10_H_12_O_2_	164.21	0.58
9	18.52	Decanoic acid, ethyl ester	C_12_H_24_O_2_	200.32	0.18
10	25.43	Hexanedioic acid, bis(2-methylpropyl)ester	C_14_H_26_O_4_	258.35	0.40
11	29.39	Dibutyl phthalate	C_16_H_22_O_4_	278.35	1.78

**Table 4:** GC-MS identification and relative content of aromatic components from bound compounds with enzymolysis.

Number	Retention time (min)	Chemical constituent	Formula	Molecular weight	Relative content (%)
1	3.18	1-Butanol, 3-methyl-, formate	C_6_H_12_O_2_	116.16	0.75
2	3.43	3-Pentanol	C_5_H_12_O	88.15	0.25
3	3.57	Propanoic acid, 2-hydroxy-, ethyl ester	C_5_H_10_O_3_	118.13	38.7
4	3.97	Furfural	C_5_H_4_O_2_	96.09	0.12
5	4.28	3-Methyl-butanoic acid	C_5_H_10_O_2_	102.13	1.29
6	4.51	2-Methyl-butanoic acid	C_5_H_10_O_2_	102.13	1.07
7	4.62	1-Hexanol	C_6_H_14_O	102.18	0.86
8	4.74	1-Butanol, 3-methyl-, acetate	C_7_H_14_O_2_	130.18	3.21
9	4.79	1-Butanol, 2-methyl-, acetate	C_7_H_14_O_2_	130.18	0.51
10	5.58	Butyrolactone	C_4_H_6_O_2_	86.09	0.16
11	6.06	Butanoic acid, 3-hydroxy-, ethyl ester	C_6_H_12_O_3_	132.15	0.25
12	7.47	Hexanoic acid	C_6_H_12_O_2_	116.16	1.93
13	7.67	Hexanoic acid, ethyl ester	C_8_H_16_O_2_	76.10	1.16
14	8.73	Benzyl alcohol	C_7_H_8_O	108.13	0.41
15	11.03	Phenylethyl alcohol	C_8_H_10_O	122.16	31.06
16	12.72	Butanedioic acid, diethyl ester	C_8_H_14_O_4_	174.20	4.06
17	12.80	Octanoic acid	C_8_H_16_O_2_	144.21	0.78
18	13.16	Octanoic acid, ethyl ester	C_10_H_20_O_2_	172.26	1.22
19	13.22	Dodecane	C_12_H_26_	170.34	0.35
20	14.87	Acetic acid, 2-phenylethyl ester	C_10_H_12_O_2_	164.21	0.33
21	18.48	Decanoic acid, ethyl ester	C_12_H_24_O_2_	200.32	0.27
22	23.33	Dodecanoic acid, ethyl ester	C_14_H_28_O_2_	228.37	0.29
23	25.39	Hexanedioic acid, bis(2-methylpropyl)ester	C_14_H_26_O_4_	258.35	0.38
24	29.33	Dibutyl phthalate	C_16_H_22_O_4_	278.35	0.72
25	31.72	Hexadecanoic acid, methyl ester	C_17_H_32_O_2_	270.00	0.26
26	33.63	9,12-Octadecadienoic acid, methyl ester	C_19_H_34_O_2_	294.47	0.48

The use of *β*-glycosidase increased considerably the total level of volatiles in wines compared to the controls. Figure 2 and Table 4 report that more aromatic components were released from the enzymolysis wine compared with the control wine. The aromatic components include 1-butanol, 3-methyl-, formate (0.75%), 3-pentanol (0.25%), furfural (0.12%), 3-methyl-butanoic acid (1.29%), 2-methyl-butanoic acid (1.07%), butanoic acid, 3-hydroxy-, ethyl ester (0.25%), hexanoic acid (1.93%), hexanoic acid, ethyl ester (1.16%), benzyl alcohol (0.41%), octanoic acid (0.78%), octanoic acid, ethyl ester (1.22%), and dodecanoic acid, ethyl ester (0.29%).

### Kramer sensory evaluation

Kramer sensory evaluation is one of the sensory-analysis methods using ranking. In this method, samples are ranked according to the degree of a specific attribute or liking. Kramer test is used to analyze the data of ranking and to identify the significant differences between samples. Three wines were presented at each session, in coded standard wine tasting glasses according to standard (ISO 3591, 1997) and covered with a watch glass to minimize the escape of volatile components. Testing temperature was 10°C. The panelists arranged the order of wines by aromatic intensity level. The strongest level was “aroma strongly perceptible” and the weakest level was “aroma not perceptible.” When there are no differences between different samples, the same bit-level was indicated in the evaluation table.

The Kramer sensory evaluation results are shown in Table 5. The 11 panelists had a very good sort for control wine samples and enzyme-treated wines. The rank and rank sum of 10 wine samples are shown in Table 6 according to Kramer sensory evaluation method. According to the table (Sun et al. 1999), the upper range was 32–89 and the lower range was 39–82 (*P *< 0.01). The 10 samples were significantly different from each other (*P *< 0.01) due to Rimax < R_F_ = 105 and Rimin > R_A_ = 25. The C, E, and B were as a group owing to 39 < R_C_ < R_E_ < R_B_ < 82. The F, G, and N were as a group owing to R_F_ > R_G_ > R_N_ > 82. The A, P, D, and O were as a group owing to R_A_ = R_P_ < R_D_ = R_O_ < 39. Enzyme-treated wines were found to be more intense in lime, honey, and smoky attributes than control wines. Similar descriptors were found on hydrolysis of glycosidase from Emir wine (Cabaroglu et al. 2003).

**Table 5:** Kramer sensory evaluation result (from weak to strong).

Evaluator	1	2	3	4	5	6	7	8	9	10
1	P	C	A	D	O	E	B	N	G	F
2	P	D	O	A	C	N	E	B	F	G
3	A	P	C	O	D	B	E	N	G	F
4	O	A	D	C	E	B	G	A	F	N
5	D	A	P	O	C	B	N	E	G	F
6	O	P	A	C	D	B	E	G	N	F
7	P	A	O	D	C	N	B	E	F	G
8	D	A	P	C	O	N	E	B	G	F
9	A	C	P	D	O	E	N	B	G	F
10	P	O	D	A	C	N	B	G	E	F
11	A	O	D	C	P	E	G	F	B	N

**Table 6:** Rank and rank sum of 10 samples of grape wine.

Evaluator	P	C	A	D	O	E	B	N	G	F
1	1	2	3	4	5	6	7	8	9	10
2	1	5	4	2	3	7	8	6	10	9
3	2	3	1	5	4	7	6	8	9	10
4	3	4	2	3	1	5	6	10	7	9
5	3	5	2	1	4	8	6	7	9	10
6	2	4	3	5	1	7	6	9	8	10
7	1	5	2	4	3	8	7	6	10	9
8	3	4	2	1	5	7	8	6	9	10
9	3	2	1	4	5	6	8	7	9	10
10	1	5	4	3	2	9	7	6	8	10
11	5	4	1	3	2	6	9	10	7	8
Rank sum	25	43	25	35	35	76	78	83	95	105

### The optimization of enzymolysis regulation conditions of wine

The analysis results are shown in Table 7 according to the rank sum of Kramer sensory evaluation. The effecting order of enzymolysis regulation conditions was enzymolysis temperature > enzymolysis time > enzyme amount. The results showed that the optimal conditions were A_2_B_3_C_1_, namely temperature of 45°C, enzymolysis time of 90 min with enzyme amount of 58.32 U/mL grape wine.

**Table 7:** Experiment result of L_9_(3^4^) orthogonal design and range analysis.

Number	Temperature (°C)	Time (min)	Enzyme volume (mL)	Sample code	Rank sum
1	1 (40)	1 (30)	1 (58.32)	B	78
2	1 (40)	2 (60)	2 (72.9)	C	43
3	1 (40)	3 (90)	3 (87.48)	D	35
4	2 (45)	1 (30)	2 (72.9)	E	76
5	2 (45)	2 (60)	3 (87.48)	F	105
6	2 (45)	3 (90)	1 (58.32)	G	95
7	3 (50)	1 (30)	3 (87.48)	P	25
8	3 (50)	2 (60)	1 (58.32)	O	35
9	3 (50)	3 (90)	2 (72.9)	N	83
K_1_	156	179	208		
K_2_	276	183	202		
K_3_	143	213	165		
R	44.3	11.3	14.3		

## Conclusion

This article contributes to the interest of the use of exogenous *β*-glycosidase in wine for enhancement of wine aroma through hydrolysis of glycosidic precursors. Our results emphasized that the optimum enzymolysis condition was: temperature at 45°C, enzymolysis time for 90 min, and enzyme addition amount of 58.32 U/mL grape wine. Compared with the control grape wine sample, the enzyme-treatment wines, which were extracted by SDE, were analyzed by GC-MS, and the yield of aromatic compounds was more: 3-methyl-1-butanol formate, 3-pentanol, furfural, 3-methyl-butanoic acid, 2-methyl-butanoic acid, 3-hydroxy-butanoic acid ethyl ester, hexanoic acid, hexanoic acid ethyl ester, benzyl alcohol, octanoic acid, octanoic acid ethyl ester, dodecanoic acid, ethyl ester, and so on. The results obtained provide a reliable indication of the aroma potential of the wines. This assay will thus be valuable when taking decisions on the use of enzyme treatments (dose, duration) applied to enhance aroma release.
